# Unveiling the noncanonical activation mechanism of CDKs: insights from recent structural studies

**DOI:** 10.3389/fmolb.2023.1290631

**Published:** 2023-11-09

**Authors:** Tao Li, Hui-Chi Tang, Kuang-Lei Tsai

**Affiliations:** ^1^ Department of Biochemistry and Molecular Biology, McGovern Medical School, University of Texas Health Science Center at Houston, Houston, TX, United States; ^2^ MD Anderson Cancer Center UTHealth Graduate School of Biomedical Sciences, Houston, TX, United States

**Keywords:** CDK7, CDK8, MED12, kinase, T-loop, Y-loop, noncanonical activation, transcription

## Abstract

The Cyclin-dependent kinases (CDKs) play crucial roles in a range of essential cellular processes. While the classical two-step activation mechanism is generally applicable to cell cycle-related CDKs, both CDK7 and CDK8, involved in transcriptional regulation, adopt distinct mechanisms for kinase activation. In both cases, binding to their respective cyclin partners results in only partial activity, while their full activation requires the presence of an additional subunit. Recent structural studies of these two noncanonical kinases have provided unprecedented insights into their activation mechanisms, enabling us to understand how the third subunit coordinates the T-loop stabilization and enhances kinase activity. In this review, we summarize the structure and function of CDK7 and CDK8 within their respective functional complexes, while also describing their noncanonical activation mechanisms. These insights open new avenues for targeted drug discovery and potential therapeutic interventions in various diseases related to CDK7 and CDK8.

## Introduction

Cyclin-dependent kinases (CDKs) are serine/threonine protein kinases that primarily phosphorylate and regulate key proteins involved in cell cycle progression, gene transcription, and other cellular processes (Malumbres, 2014). The members of the CDK family in metazoans can be broadly categorized into two sets based on their primarily functions within the cell: 1) the cell cycle-related CDKs, such as CDK1, CDK2, CDK4 and CDK6, which play a crucial role in regulating the progression of the cell cycle (Matthews et al., 2022); 2) the transcriptional CDKs, including CDK7, CDK8/CDK19, CDK9, CDK11 and CDK12/CDK13, involved in a series of events during gene transcription and responsible for phosphorylating the carboxyl terminal domain (CTD) of the largest subunit RPB1 of RNA polymerase II (RNAPII) (Malumbres, 2014; Espinosa, 2019; Chou et al., 2020). It is worth noting that certain transcriptional CDKs, such as CDK7 and CDK8, can have dual functions, participating in both transcriptional processes and cell cycle regulation (Lolli and Johnson, 2005; Szilagyi and Gustafsson, 2013).

In principle, CDK monomers exhibit only minimal detectable kinase activity (Lees, 1995). For canonical CDKs, such as CDK2/CycA and CDK9/CycT, full activation requires both the binding of the cyclin partner and the phosphorylation of a highly conserved threonine residue located in a characteristic activation segment (T-loop) (Morgan, 1995; Russo et al., 1996; Baumli et al., 2008). Intriguingly, there are a few noncanonical CDKs which require an additional subunit for their full activation. Among those, CDK7 and CDK8, conserved throughout all eukaryotes and known for their roles in regulating the transcription process, are integrated into large transcriptional complexes—the CAK (CDK-activating kinase complex) module of the TFIIH and the CDK8 kinase module (CKM) of the Mediator, respectively. The manner in which CDK7 and CDK8 are assembled into the CAK and CKM, respectively, and activated by the third subunits has remained unclear until recently. Since 2020, several structural studies focusing on eukaryotic CAK or CKM have been reported (Table 1) (Greber et al., 2020; 2021; Peissert et al., 2020; Eeuwen et al., 2021; Li et al., 2021). Here, we review these structures and summarize their functions and activation mechanisms, providing a comprehensive understanding of both noncanonical CDKs.

**TABLE 1:** Summary of the structures of noncanonical CDK complexes.

CDK kinase	Complex	Species	PDB ID	Ligand	Resolution Å)	Method	Year
CDK8	CKM (Cdk8/CycC/Med12/Med13)	*Saccharomyces cerevisiae*	7KPV	apo	3.8	Cryo-EM	2021
			7KPX	apo	4.4		
CDK7	CAK (CDK7/CycH/MAT1)	*Homo sapiens*	6XBZ	ATP-γ-S	2.8	Cryo-EM	2020
			6XD3	THZ1	3.3		
	CAK (CDK7/CycH/MAT1)	*Homo sapiens*	7B5O	ICEC0942	2.5	Cryo-EM	2021
	TFIIK (Kin28/Ccl1/Tfb3)	*Saccharomyces cerevisiae*	7KUE	ADP·AF3	3.5	Cryo-EM	2021
	CAK (CDK7/CycH/MAT1)	Chaetomium thermophilum	6Z3U	apo	2.6	X-ray	2020
			6Z4X	ATP-γ-S	2.98		

## Classical two-step activation mechanism

**FIGURE 1:**
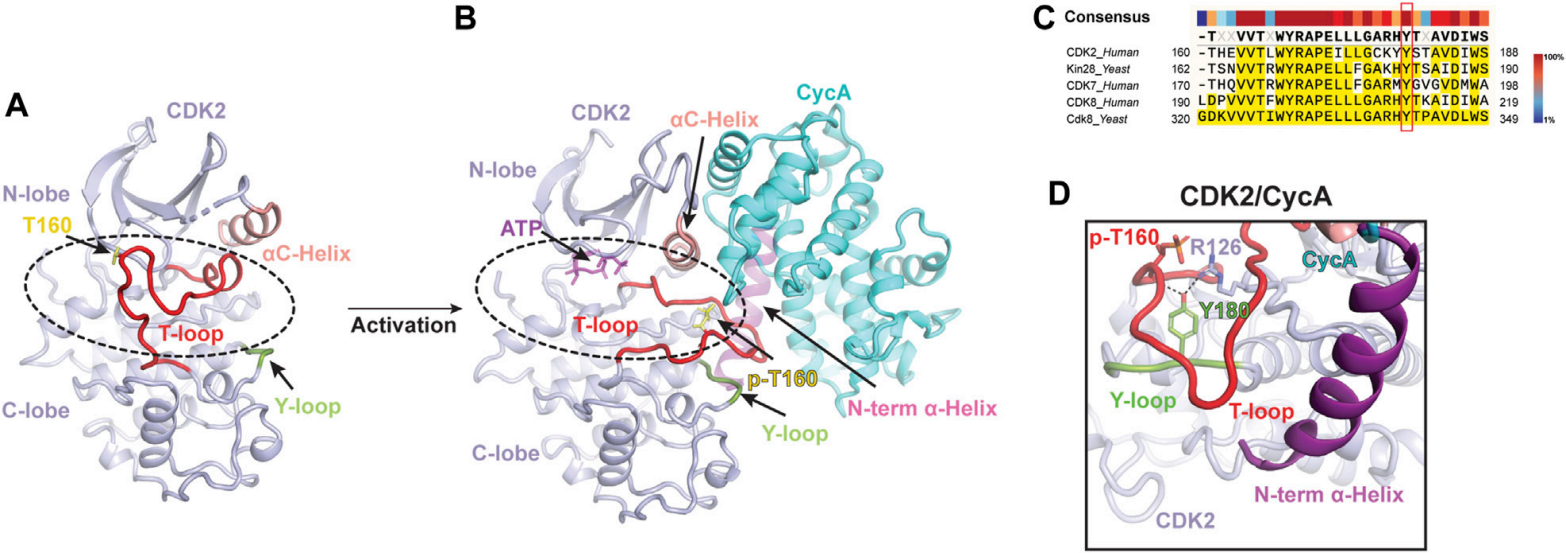
Classical two-step activation mechanism of CDK2 **(A)** Crystal structure of monomeric human CDK2 [PDB: 1HCL](Schulze-Gahmen et al., 1996). The T-loop (red) obstructs substrates access to the catalytic cleft (indicated by a dashed black oval) of CDK2 (light purple). **(B)** Crystal structure of human CDK2/CycA complex bound with ATP [PDB: 1JST](Russo et al., 1996). CycA (cyan) binding and T160 phosphorylation (yellow) release the blockade of the catalytic cleft (indicated by a dashed black oval), leading to the activation of CDK2. **(C)** Sequence alignment of the Y-loop in various CDK proteins. Conserved Tyr residues are highlighted in the red box. **(D)** Y180 of the Y-loop interacts with p-T160 and R126 of human CDK2, contributing to T-loop stabilization. Y-loop and T-loop are colored in green and red, respectively. Key residues are indicated.

Classical CDK proteins can be switched into an active state in two steps. The first step involves binding to their cyclin partner, resulting in partial activation of kinase activity. The second critical step entails the phosphorylation of the activation segment (T-loop) of CDK, which greatly enhances its kinase activity to a higher level (Morgan, 1995; Russo et al., 1996). Among the CDK family proteins, the structure and activation mechanism of CDK2, along with its complex with the cyclin partner CycA, were thoroughly studied (Figure 1). The CDK2 structure consists of a *ß*-sheet-rich N-terminal lobe and a compact C-terminal lobe, which encloses a catalytic cleft responsible for ATP binding and substrate recognition. The T-loop in monomeric CDK2 adopts an inactive conformation, which obstructs the catalytic cleft and renders substrates inaccessible (Figure 1A) (Bondt et al., 1993; Schulze-Gahmen et al., 1996). This explains why monomeric CDK2 displays minimal activity.

To activate the kinase function of CDK2, a series of interactions are required to stabilize the T-loop in an active conformation (Figure 1B). First, the CycA, composed of two cyclin-fold boxes (CFB), binds to the catalytic cleft of CDK2, inducing conformational changes in its αC-helix (the PSTAIRE helix) and T-loop. The αC-helix is shifted closer to the ATP-binding site, which, subsequently, directs the inactive T-loop away from the entrance of the cleft. Thus, the CycA binding alleviates the steric blockade of the catalytic cleft, which partially activates the kinase function of CDK2 (Jeffrey et al., 1995; Schulze-Gahmen et al., 1996). Additionally, this conformational change also re-orientates the aspartate residue (D145) of the DFG motif into a “DFG-in” state, in which the D145 sidechain coordinates a magnesium ion for ATP binding (Jeffrey et al., 1995). Second, the full kinase activity of CDK2 is achieved by the phosphorylation of T160 (p-T160), a highly conserved threonine residue on the T-loop throughout the entire CDK family (Russo et al., 1996). The phosphate group of p-T160 functions as a re-organization center, reshaping the T-loop through interactions with three conserved arginine sidechains (R50, R126 and R150), positioned at the N-lobe, C-lobe, and T-loop, respectively. Besides these residues, a conserved tyrosine residue (Y180 in human CDK2) (Figure 1C), positioned within a short loop (referred to as Y-loop) between two conserved helices adjacent to the T-loop, undergoes repositioning to participate in the interaction network mediated by p-T160 (Figures 1A, B, D). Upon phosphorylation on T160, the tip of the T-loop is further stabilized through numerous contacts with CycA, including additional interactions with the N-terminal α-helix of CycA (Figure 1B). This further extends the T-loop, shaping it into a platform suitable for substrate binding. All these conformational rearrangements upon CycA binding and T-loop phosphorylation render the catalytic cleft accessible, resulting in a fully active CDK2 (Russo et al., 1996; Pavletich, 1999). This two-step activation mechanism is considered a fundamental principle for classical CDKs (Figure 1B) (Morgan, 1995; Pavletich, 1999).

Among the transcriptional CDKs, CDK9 has been subject to thorough investigation. Together with Cyclin T, it forms the positive transcription elongation factor b (P-TEFb), which plays a pivotal role in reversing elongation repression by phosphorylating the Ser2 of RNAPII CTD, the hSPT5 subunit of DSIF and the NELF-E/RD subunit of NELF (Yamaguchi et al., 1999; Fujinaga et al., 2004; Yamada et al., 2006). Notably, CDK9 follows the classical two-step activation mechanism, initially interacting with Cyclin T and then undergoing T186 phosphorylation (Baumli et al., 2008). Interestingly, two other transcriptional CDKs, CDK7 and CDK8, are integrated into large functional complexes, which require the participation of additional protein subunits for their full activation. In this context, we comprehensively review and summarize the structural features and noncanonical activation mechanisms of these two transcriptional CDKs (CDK7 and CDK8), as described below.

## Mediator and its dissociable CKM

The eukaryotic Mediator is a multisubunit (25–30 proteins) transcriptional coactivator complex that transmits regulatory signals from activators and repressors to the RNAPII transcription machinery (Malik and Roeder, 2010; Soutourina, 2018). This complex consists of a large Core (cMED) and a dissociable kinase module, encompassing CDK8, CycC, MED12, and MED13. Initially, Cdk8, CycC, Med12 and Med13 were discovered in yeast as Srb10, Srb11, Srb8 and Srb9, respectively, and copurified with other Srb proteins in the RNA polymerase II (RNAPII) holoenzyme (Hengartner et al., 1995; Liao et al., 1995). Subsequently, a heterotetrameric subcomplex, consisting of Srb10 (Cdk8), Srb11 (CycC), Srb8 (Med12), and Srb9 (Med13), was isolated from yeast and has been identified across all eukaryotes (Borggrefe et al., 2002). In humans, CDK8 (initially identified as K35 (Tassan et al., 1995b)) and CycC, together with MED12 and MED13, originally known as TRAP230/ARC240 and TRAP240/ARC250 (Ito et al., 1999; Samuelsen et al., 2003), respectively, constitute a 600 kDa protein complex, called CDK8 kinase module (CKM) (Taatjes et al., 2002; Knuesel et al., 2008; 2009; Allen and Taatjes, 2015). Additionally, vertebrate paralogs of MED12, MED13, and CDK8 have also been identified and designated as MED12L, MED13L and CDK19, respectively. Each of the paralogs is found to be mutually exclusive of one another, yet is not exclusive to any other members of CKM. These paralogs are believed to have specialized roles in cellular processes (Clark et al., 2015). The CKM regulates gene transcription through both kinase-independent and kinase-dependent mechanisms. It has been demonstrated that CKM can reversibly interact with cMED through MED13, potentially hindering the binding of RNAPII to cMED. Nevertheless, the precise molecular mechanism underlying this phenomenon remains poorly understood.

## Positive and negative roles of CDK8 in gene transcription

Studies have demonstrated that CDK8 within the CKM possesses the capacity to phosphorylate the CTD of RNAPII at Ser2 and Ser5 *in vitro* (Rickert et al., 1999; Borggrefe et al., 2002; Donner et al., 2010). The phosphorylation of CTD by yeast Cdk8/CycC impedes the formation of the pre-initiation complex (Hengartner et al., 1998; Knuesel et al., 2009). Furthermore, Cdk8 phosphorylates CycH within TFIIH, thereby obstructing transcription initiation (Akoulitchev et al., 2000). Based on these initial studies, Cdk8 was primarily attributed a negative regulatory role in transcription. However, subsequent investigations have unveiled its additional capability for positive transcriptional regulation. In the Wnt/β-catenin pathway, p53 target genes, and TGF-β pathways, CDK8 has been shown to play a positive regulatory role (Donner et al., 2007; Firestein et al., 2008; Alarcón et al., 2009). In addition, the CDK8-Mediator complex is considered as a positive regulator of transcriptional elongation, facilitating release of RNAPII from the paused state and promoting transcription elongation during serum and hypoxia responses (Donner et al., 2010; Galbraith et al., 2013). CDK8 also activates IFN-γ pathway by phosphorylating the STAT1 transcription factor (Steinparzer et al., 2019). Notably, a range of human CDK8/19 substrates, including DNA-binding transcription factors and chromatin associated proteins, has been identified in human cells, showing that the CDK8 plays diverse regulatory roles in various cellular processes (Poss et al., 2016; Barron et al., 2021).

## CDK8/CycC structure with an inactive T-loop

**FIGURE 2:**
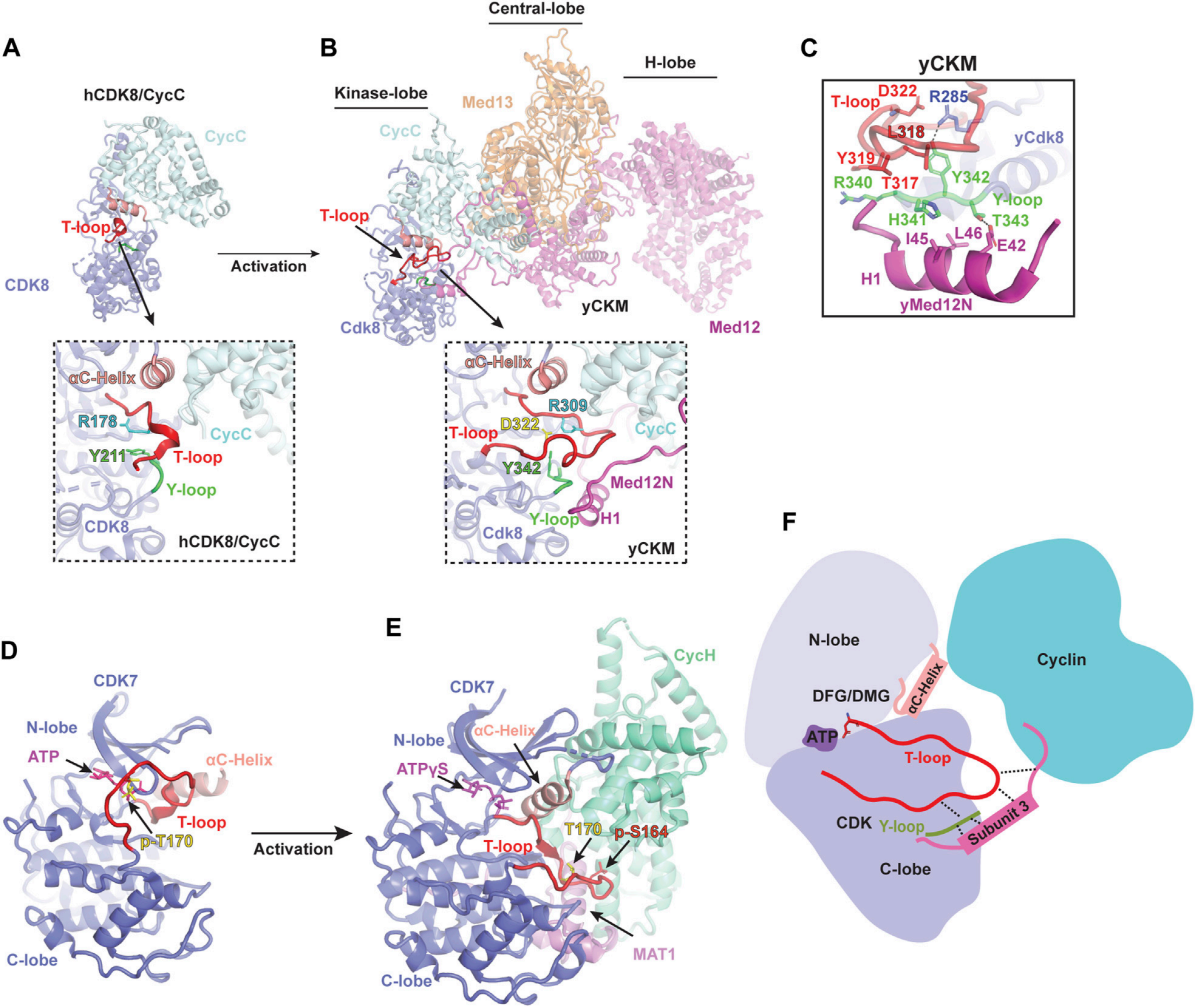
Activation mechanism of noncanonical CDKs **(A)** Crystal structure of human CDK8/CycC heterodimer in apo state [PDB: 4G6L](Schneider et al., 2013). CDK8 and CycC are colored in purple and cyan, respectively. Only a small portion of the T-loop density was observed, indicating an inactive state even when bound by CycC. The detailed conformation of the catalytic cleft is zoomed in below. **(B)** Cryo-EM structure of yeast Cdk8 kinase module (CKM) [PDB: 7KPX](Li et al., 2021). Subunits of CKM (Cdk8/CycC/Med12/Med13) are colored and labeled individually. The T-loop (red) is well-defined in an active conformation (zoomed in below). **(C)** Interactions of the Y-loop with the H1 helix of Med12N to stabilize the T-loop. Y-loop and T-loop are colored in green and red, respectively. Key residues are indicated. **(D)** Crystal structure of monomeric human CDK7 in complex with ATP [PDB: 1UA2](Lolli et al., 2004). CDK7 is shown in purple with the T-loop colored in red. The T-loop in monomeric CDK7 obstructs the catalytic cleft, preventing substrate binding even when T170 is phosphorylated (yellow). **(E)** Cryo-EM structure of human CDK-activating kinase complex (CAK) bound with ATPγS [PDB: 6XBZ](Greber et al., 2020). The CDK7, CycH and MAT1 of the CAK are colored in purple, green and magenta, respectively. The activated T-loop (red) adopts an extended conformation stabilized by contacts through MAT1 and CycH. **(F)** Schematic diagram illustrating the activation mechanism of noncanonical CDKs. Firstly, cyclin binding pushes the αC-helix of CDK towards the ATP-binding site, causing the T-loop to move away from the entrance of the catalytic pocket. Secondly, the third subunit stabilizes the T-loop by making contacts with its tip and binding to the Y-loop, which subsequently interacts with a conserved arginine residue from the C-lobe of CDK.

The first structure of human CDK8/CycC in complex with an inhibitor, Sorafenib, was determined by X-ray crystallography (Schneider et al., 2011). Similar to CDK2, the structure of CDK8 consists of an N-lobe and a C-lobe, between which is the catalytic cleft. Notably, CDK8 contains an additional N-terminal αB-helix, which is responsible for recognizing CycC (Figure 2A). The CycC subunit shares two general cyclin-fold boxes (N-CFB and C-CFB), but exclusively contacts the N-lobe of CDK8. The CycC binding prompts CDK8 to adopt an “αC-helix pushed-in” conformation. Due to the small molecule, Sorafenib, bound to the catalytic cleft, CDK8 adopts a “DMG-out” (DLG in yeast Cdk8) state (Schneider et al., 2011). To date, numerous crystal structures of human CDK8/CycC complex have been determined, including the apo state and its complexes with various inhibitors, such as Cortistatin A, and CCT251545 (Dale et al., 2015; Pelish et al., 2015; Xi et al., 2019). Intriguingly, all of the T-loops within these structures are partially absent, indicating an inactive state of CDK8 even when bound by CycC (Figure 2A). Additionally, what distinguishes CDK8 from other CDKs is the absence of the conserved threonine within its T-loop, which is typically phosphorylated in other activated CDKs. Without the phosphorylated residue, the sidechain of the conserved tyrosine Y211 in human CDK8 Y-loop adopts an orientation that might potentially clash with the T-loop (Figure 2A). The collision could hinder the formation of the stabilized and activated T-loop. This could provide an explanation for the observed disorder or inactive conformation of the T-loops within human CDK8/CycC structures, resulting in the obstruction of the catalytic cleft. This observation aligns with the notion that human CDK8/CycC displays minimal kinase activity in the absence of MED12 (Knuesel et al., 2008; Turunen et al., 2014; Park et al., 2018).

## Structure of CKM

Dysfunction or dysregulation of CKM subunits has been extensively linked to many human diseases (Clark et al., 2015). Among the CKM subunits, CDK8 has been recognized as a potent oncogene in colorectal cancer (Firestein et al., 2008), making it a promising therapeutic target. While structural information regarding the inactivation of human CDK8 when bound by inhibitors is available, our understanding of its activation mechanism at molecular level remains limited. In 2021, the cryo-EM structure of the yeast CKM (500 kDa) was reported, providing the first detailed structural information regarding its architecture and subunit interaction (Li et al., 2021). The overall structure of the CKM is organized into three lobes: the Kinase-, Central-, and H-lobe (Figure 2B). In the Kinase-lobe, the Med12N, consisting of two α-helices (H1 and H2) and two coils, establishes extensive contacts with Cdk8 and CycC. The Central-lobe consists of Med13 and the HEAT-I and C-terminal segment of Med12, while the H-lobe forms a large horseshoe-shaped solenoid structure (HEAT I to IV of Med12). Med12, consisting of the N-terminal segment (Med12N), a large horseshoe-shaped solenoid structure (Med12HEAT) and C-terminal segment (Med12C), functions as a scaffold connecting the three lobes and providing a large surface for transcription regulators. Interestingly, Med13 possesses an Argonaute-like structure, indicating its function related to interaction with nucleic acids.

## Noncanonical activation mechanism of CDK8

Within yeast CKM, the structure of Cdk8/CycC closely resembles that of human CDK8/CycC (Figure 2B) (Li et al., 2021). Interestingly, a well-defined Cdk8 T-loop in the catalytic cleft was observed for the first time. A series of interactions involving Y342 of the Y-loop and three conserved arginine residues R206, R285 and R309 (corresponding to R65, R150 and R178 in human CDK8, respectively), as well as the Med12N, are established to stabilize the T-loop in an active conformation. Due to the lack of the conserved phosphorylated threonine in the Cdk8 T-loop, Med12N binding helps adjust the Y342 within the Y-loop to interact with R285, thus contributing to the T-loop stabilization (Figure 2C). Moreover, the Med12N also forms contacts with the tip of the T-loop and the Y-loop of Cdk8, contributing more interactions for T-loop stabilization (Figure 2B). Differing from the configuration observed in the yeast CKM, where R309 undergoes repositioning to mitigate this hindrance, R178 residue in human CDK8/CycC within the T-loop is situated at the entrance of the catalytic pocket, potentially hindering its accessibility (Figure 2A) (Schneider et al., 2013). Thus, Med12N binding supersedes the conserved phosphorylated threonine residue for stabilizing the T-loop.

Due to high sequence similarity between yeast and human, the human MED12N was predicted to contain an “activation helix” (residues 32–44) in proximity to the T-loop of CDK8. The acidic triad (E33-D34-E35) at the beginning of this activation helix is responsible for positioning it properly for CDK8 activation (Klatt et al., 2020). This acidic triad EDE is conserved in yeast Med12N (E42-D43-Q44), which are located exactly at the H1 Helix, fostering the stabilization of the T-loop. These findings not only suggest that the regulatory principles governing CDK8 activation are conserved between yeast and human, but also underscore the functional relevance and significance of Med12 in regulating CDK8 activity across various species. Additionally, the yeast CKM structure provides structural insights into the reason behind hotspot mutations in MED12 (G44D, L36R, Q43P, and G44S), which are associated with Uterine Leiomyoma and situated near the T-loop (Turunen et al., 2014). These mutations disrupt the interaction of MED12 with the CDK8/CycC dimer, consequently leading to diminished kinase activity (Turunen et al., 2014; Klatt et al., 2020; Li et al., 2021). This insight offers a fresh avenue strategy for drug development that can focus on interrupting the interface between the MED12 and CDK8. The yeast CKM structure not only illuminates a comprehensive understanding of noncanonical CDK8 activation but also establishes a structural foundation for comprehending the function of CKM and its role in pathological dysfunction. Further comprehension is required regarding how substrates bind to the catalytic pocket in the presence of MED12, the deactivation process of CDK8, and whether the human CKM possesses a more intricate structure and additional regulatory functions.

## CDK7: requirement of MAT1 to assemble the CAK complex

CDK7 was initially discovered in *Xenopus* as MO15, and its homologues were later identified in mammals and named as CDK7 (Shuttleworth et al., 1990; Fisher and Morgan, 1994; Shuttleworth, 1995). The cyclin partner of CDK7 was found to be CycH (Fisher and Morgan, 1994; Mäkelä et al., 1994). In contrast to most cell cycle-related CDKs, CDK7/CycH requires an additional subunit, the RING finger protein MAT1, to form an active ternary CDK-activating kinase (CAK) complex (Tassan et al., 1995a; Devault et al., 1995). The CAK plays a vital role in activating and phosphorylating cell cycle-related CDKs, such as CDK1, CDK2, CDK4 and CDK6 (Fesquet et al., 1993; Poon et al., 1993; Matsuoka et al., 1994; Jeffrey et al., 2000). As a dual functional kinase, the CAK also forms part of the general transcription factor TFIIH, a 10-subunit complex composed of a large Core and a CDK7/CycH pair connected through the MAT1 (Roy et al., 1994; Serizawa et al., 1995; Shiekhattar et al., 1995; Compe and Egly, 2012). CDK7, requiring both the binding of CycH and the C-terminus of MAT1 for kinase activation, phosphorylates Ser5 and Ser7 of the RNAPII CTD during transcription process (Gileadi et al., 2000; Akhtar et al., 2009; Glover-Cutter et al., 2009; Fisher, 2019).

Several studies have predominantly focused on unraveling the structures of the Core of the TFIIH and CDK7 (Lolli et al., 2004; He et al., 2016; Greber et al., 2017; 2019; Schilbach et al., 2017; Yan et al., 2019; Chen et al., 2022; Abril-Garrido et al., 2023; Wang et al., 2023), leaving a gap in knowledge regarding CDK7 activation until recently. The first crystal structure of human CDK7 bound with ATP was reported in 2004 (Lolli et al., 2004), revealing a typical CDK kinase fold with the catalytic pocket positioned between the N-terminal and C-terminal lobes (Figure 2D). Although the ATP-binding site of CDK7 is accessible, its substrate-binding site remains hindered by the inactive T-loop, leading to the lack of kinase activity (Lolli et al., 2004). While the structure of CDK7/CycH heterodimer remains unavailable, substantial progress has been made in the past 3 years regarding the structure determination of CAK across various species, including human, budding yeast, and Chaetomium thermophilum (Ct) (Table 1) (Greber et al., 2020; 2021; Peissert et al., 2020; Eeuwen et al., 2021). These advancements offer valuable insights into the mechanism of CDK7 activation (Figure 2D) (Greber et al., 2020; 2021; Peissert et al., 2020; Eeuwen et al., 2021).

## Activation mechanism of CDK7

In the human CAK structure, the αC-helix of the CDK7 is shifted toward the catalytic site, and the T-loop adopts an active conformation (Figure 2E) (Greber et al., 2020). Interestingly, the CDK7 within the CAK is in an active state, even though the conserved threonine residue (T170) on the T-loop remains unphosphorylated (Fisher et al., 1995). The tip of the activated T-loop is in extensive contacts with a C-terminal α-helix from MAT1. This helix extends along the groove between the C-lobe of CDK7 and the C-CBF of CycH. Notably, the positioning of this MAT1 α-helix is reminiscent of the N-terminal α-helix of CycA, where it interacts with the tip of the T-loop of CDK2 (Figure 2E) (Jeffrey et al., 1995). Additionally, human CDK7 harbors an additional phosphorylation residue, S164 (p-S164), located within its T-loop. The p-S164 is in proximity to a positively charged pocket formed by three arginine residues from each subunit of the CAK complex (R167, R295, and R165 from CDK7, MAT1, and CycH, respectively). This interaction network contributes to the assembly of the complex and potentially stabilizes the T-loop of human CDK7 (Greber et al., 2020).

The budding yeast counterpart of the human CAK complex is known as TFIIK, comprising Kin28, Ccl1, and Tfb3 (equivalent to CDK7, CycH, and MAT1, respectively) (Feaver et al., 1994; 1997; Keogh et al., 2002). In the TFIIK structure, the T-loop of Kin28 is phosphorylated and adopts an active conformation (Eeuwen et al., 2021). In this context, the tip of the T-loop is further stabilized through hydrophobic interactions involving both the C-terminal α-helix and the short 3_10_ helix of Tfb3. Notably, the C-terminal α-helix of Tfb3 is positioned similarly to the N-terminal α-helix of CycA, allowing it to interact with the tip of the T-loop. On the other hand, the CtCAK utilizes a similar manner to stabilize the tip of the T-loop (Peissert et al., 2020). A conserved P248 on the CtCDK7 T-loop points into a hydrophobic pocket formed by the C-terminal α-helix and its adjacent short helix of CtMAT1. Moreover, its D247, corresponding to the phosphorylated S164 of human CDK7, interacts CtCycH and CtMAT1, contributing to T-loop stabilization. Based on these CAK structures, the C-terminal region of MAT1/Tfb3 binding is necessary for T-loop stabilization (Figure 2E). The role of MAT1/Tfb3 is similar to that of the Med12 N-terminus which contributes to the noncanonical activation of Cdk8. CDK7 plays multiple crucial roles in cell-cycle control and gene transcription. Therefore, these structures of the CAK complex reveal how it assembles and becomes active, shedding light on the molecular mechanism of CDK7 activation and providing detailed information for the design of therapeutic compounds targeted on CDK7.

## Rearrangement of the Y-loop facilitates T-loop stabilization

The inactive T-loop in monomeric CDK adopts a folded conformation, which hinders the catalytic cleft (Figure 1A) (Bondt et al., 1993; Schulze-Gahmen et al., 1996). To activate the kinase, a series of interactions are required to stabilize the T-loop in an active conformation. Significantly, a highly conserved tyrosine residue (Y180 in human CDK2) in the Y-loop of CDK plays an important role during this process (Figure 1C). In the structure of CDK2/CycA complex, the Y180 in the Y-loop is involved in T-loop stabilization by contacting with both R126 of CDK2 and T160 (Figure 1D) (Russo et al., 1996). Disruption of these interactions by a peptide can inhibit the kinase activity of CDK2, supporting the significance of Y180 of the Y-loop in the activation of CDK kinases (Chen et al., 2014).

Interestingly, among the CDKs, CDK8 stands alone in lacking the conserved threonine residue on its T-loop. This absence suggests the utilization of an alternative mechanism to rearrange the Y342 sidechain in order to interact with the conserved arginine residues. In the CKM structure, the N-terminal H1 helix of Med12 interacts with two residues (H341 and T343) of the Y-loop, leading to repositioning of the Y342 sidechain (Li et al., 2021). This adjustment facilitates interaction with R285 of yeast Cdk8, thus creating a specific interaction network crucial for T-loop stabilization (Figure 2C). This Y-loop rearrangement induced by MED12 clarifies the necessity of MED12 for CDK8 activation (Figure 2B). Similarly, in the structures of CAK and TFIIK, the Y-loop of CDK7/Kin28 also makes contacts with both MAT1/Tfb3 and the conserved R2 residue (Greber et al., 2020; Eeuwen et al., 2021). This illustrates that the Y-loop of noncanonical CDK functions as a bridge to facilitate the T-loop stabilization. In addition to their interaction with the Y-loop, both MED12N and MAT1 also establish direct contacts with the tip of the T-loop, enhancing T-loop stabilization and overall complex assembly. As a result, they assume a vital role in the activation of noncanonical kinases (Figure 2F).

## Concluding remarks

Several canonical CDK proteins have been extensively investigated through functional and structural studies, revealing the essential role of both cyclin binding and T-loop phosphorylation in achieving a fully active state (Schulze-Gahmen and Kim, 2002; Baumli et al., 2008; Bösken et al., 2014; Wood and Endicott, 2018). However, this classical two-step mechanism falls short in explaining the activation of two transcriptional kinases, CDK7 and CDK8. Each of these kinases is a component of the large TFIIH and CKM complexes (Serizawa et al., 1995; Shiekhattar et al., 1995; Taatjes et al., 2002; Tsai et al., 2013), respectively, and requires an additional step to attain full activation. Recent structural studies on these transcriptional kinase complexes have unveiled their noncanonical activation mechanisms. In the case of CDK7 activation, along with CycH binding and T-loop phosphorylation, MAT1 establishes extensive contacts with both T- and Y-loops, resulting in a stabilized T-loop that releases the hindrance in the catalytic cleft (Greber et al., 2020; Peissert et al., 2020; Eeuwen et al., 2021). As for CDK8, lacking a phosphorylation residue on T-loop, MED12 plays a crucial role in establishing contacts with both T- and Y-loops (Li et al., 2021). This effectively replaces the function of the conserved phosphorylated threonine residue to stabilize the T-loop. Within large complexes, these precise activation processes ensure the accessibility of the ATP binding pocket and substrate binding site, enabling the kinase to proficiently phosphorylate its substrates. These novel findings pave the way for targeted approaches in drug discovery to tackle CDK8 and CDK7-related diseases, bring us closer to the development of effective therapeutics.
